# Feature-Free Activity Classification of Inertial Sensor Data With Machine Vision Techniques: Method, Development, and Evaluation

**DOI:** 10.2196/mhealth.7521

**Published:** 2017-08-04

**Authors:** Jose Juan Dominguez Veiga, Martin O'Reilly, Darragh Whelan, Brian Caulfield, Tomas E Ward

**Affiliations:** ^1^ Insight Centre for Data Analytics Department of Electronic Engineering Maynooth University Maynooth Ireland; ^2^ Insight Centre for Data Analytics University College Dublin Dublin Ireland

**Keywords:** machine learning, exercise, biofeedback

## Abstract

**Background:**

Inertial sensors are one of the most commonly used sources of data for human activity recognition (HAR) and exercise detection (ED) tasks. The time series produced by these sensors are generally analyzed through numerical methods. Machine learning techniques such as random forests or support vector machines are popular in this field for classification efforts, but they need to be supported through the isolation of a potentially large number of additionally crafted features derived from the raw data. This feature preprocessing step can involve nontrivial digital signal processing (DSP) techniques. However, in many cases, the researchers interested in this type of activity recognition problems do not possess the necessary technical background for this feature-set development.

**Objective:**

The study aimed to present a novel application of established machine vision methods to provide interested researchers with an easier entry path into the HAR and ED fields. This can be achieved by removing the need for deep DSP skills through the use of transfer learning. This can be done by using a pretrained convolutional neural network (CNN) developed for machine vision purposes for exercise classification effort. The new method should simply require researchers to generate plots of the signals that they would like to build classifiers with, store them as images, and then place them in folders according to their training label before retraining the network.

**Methods:**

We applied a CNN, an established machine vision technique, to the task of ED. Tensorflow, a high-level framework for machine learning, was used to facilitate infrastructure needs. Simple time series plots generated directly from accelerometer and gyroscope signals are used to retrain an openly available neural network (Inception), originally developed for machine vision tasks. Data from 82 healthy volunteers, performing 5 different exercises while wearing a lumbar-worn inertial measurement unit (IMU), was collected. The ability of the proposed method to automatically classify the exercise being completed was assessed using this dataset. For comparative purposes, classification using the same dataset was also performed using the more conventional approach of feature-extraction and classification using random forest classifiers.

**Results:**

With the collected dataset and the proposed method, the different exercises could be recognized with a 95.89% (3827/3991) accuracy, which is competitive with current state-of-the-art techniques in ED.

**Conclusions:**

The high level of accuracy attained with the proposed approach indicates that the waveform morphologies in the time-series plots for each of the exercises is sufficiently distinct among the participants to allow the use of machine vision approaches. The use of high-level machine learning frameworks, coupled with the novel use of machine vision techniques instead of complex manually crafted features, may facilitate access to research in the HAR field for individuals without extensive digital signal processing or machine learning backgrounds.

## Introduction

### Background

Inertial sensors are ubiquitous in everyday objects such as mobile phones and wristbands and can provide large amounts of data regarding movement activity. Analysis of such data can be diverse, but in general terms can be characterized as complex operations using a broad range of machine learning techniques and highly sophisticated signal processing methods. The latter is required to extract salient features that can improve recognition performance. These features are not only complex to calculate, but also making a priori reasoned arguments toward their effectiveness in improving overall results is difficult. The temptation to include additional features in an attempt to improve classification accuracy may result in pipelines (infrastructure) with excessive complexity, yielding slower processing and increased resource usage. To counter this proliferation of features, it is common to use dimensionality reduction techniques including linear approaches such as principal component analysis and increasingly common nonlinear methods principally based on manifold learning algorithms.

In contrast to this complex tool, we propose a method to classify human activity from inertial sensor data based on images and using deep learning-based machine vision techniques. This approach reduces the amount of deep domain knowledge needed in terms of digital signaling processing (DSP), down to some basic steps of preprocessing and segmentation, substituting instead a neural network that can learn the appropriate features independent of a user-driven feature candidature step. Convolutional networks are not trivial to work with, but the recent availability of higher level deep learning frameworks such as TensorFlow [1] and the use of transfer learning, a technique to reuse already trained convolutional neural networks (CNNs), considerably reduces the skills needed to set up and operate such a network.

In this study, we sought to demonstrate a novel application of machine vision techniques as a classification method for inertial measurement unit (IMU) data. The main goal of this work was to develop a novel data analysis pathway for researchers who are most interested in this type of work, such as medical and exercise professionals. These individuals may not have the technical background to implement existing state-of-the-art data analysis pathways. We also aimed to evaluate the efficacy of our new classification technique by attempting to detect five commonly completed lower-limb exercises (squats, deadlifts, lunges, single-leg squats, and tuck jumps) using the new data analysis pathway. The accuracy, sensitivity, and specificity of the pathway were compared with recently published work on the same dataset.

### Related Work

The three main topics in this section are as follows: (1) a brief overview of the current human activity recognition (HAR) and exercise detection (ED) literature, (2) an account of some of the newer advances in the field that are using neural networks for certain parts of the feature discovery and reduction process, and (3) an introduction to transfer learning, highlighting its benefits in terms of time and resource savings, and working with smaller datasets.

#### Activity Classification for Inertial Sensor Data

Over the past 15 years, inertial sensors have become increasingly ubiquitous due to their presence in mobile phones and wearable activity trackers [2]. This has enabled countless applications in the monitoring of human activity and performance spanning applications in general HAR, gait analysis, the military field, the medical field, and exercise recognition and analysis [3-6]. Across all these application spaces, there are common challenges and steps which must be overcome and implemented to successfully create functional motion classification systems.

Human activity recognition with wearable sensors usually pertains to the detection of gross motor movements such as walking, jogging, cycling, swimming, and sleeping [5,7]. In this field of motion tracking with inertial sensors, the key challenges are often considered to be (1) the selection of the attributes to be measured; (2) the construction of a portable, unobtrusive, and inexpensive data acquisition system; (3) the design of feature extraction and inference methods; (4) the collection of data under realistic conditions; (5) the flexibility to support new users without the need for retraining the system; and (6) the implementation in mobile devices meeting energy and processing requirements [3,7]. With the ever-increasing computational power and battery life of mobile devices, many of these challenges are becoming easier to overcome.

Whereas system functionality is dependent on hardware constraints, the accuracy, sensitivity, and specificity of HAR systems are most reliant on building large, balanced, labeled datasets; the identification of strong features for classification; and the selection of the best machine learning method for each application [3,8-10]. Investigating the best features and machine learning methods for each HAR application requires an individual or team appropriately skilled in signal processing and machine learning and a large amount of time. They must understand how to compute time-domain, frequency-domain, and time-frequency domain features from inertial sensor data and train and evaluate multiple machine learning methods (eg, random forests [11], support vector machines [12], k-nearest neighbors [13], and logistical regression [14]) with such features [3-5]. This means that those who may be most interested in the output of inertial sensor based activity recognition systems (eg, medical professionals, exercise professionals, and biomechanists) are unable to design and create the systems without significant engagement with machine learning experts [4].

**Figure 1:**
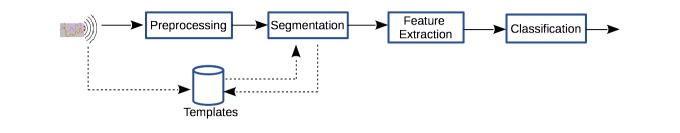
Steps involved in the development of an inertial measurement unit (IMU)-based exercise classification system.

The above challenges in system design and implementation are replicated in activity recognition pertaining to more specific or acute movements. In the past decade, there has been a vast amount of work in the detection and quantification of specific rehabilitation and strength and conditioning exercises [15-17]. Such work has also endeavored to detect aberrant exercise technique and specific mistakes that system users make while exercising, which can increase their chance of injury or decrease their body’s beneficial adaptation due to the stimulus of exercise [17,18]. The key steps in the development of such systems have been recently outlined as (1) inertial sensor data collection, (2) data preprocessing, (3) feature extraction, and (4) classification (Figure 1) [4]. Whereas the first step can generally be completed by exercise professionals (eg, physiotherapists and strength and conditioning coaches), the remaining steps require skills outside that included in the training of such experts. Similarly, when analyzing gait with wearable sensors, feature extraction and classification have been highlighted as essential in the development of each application [19,20]. This again limits the type of professional who can create such systems and the rate at which hypotheses for new systems can be tested.

#### Neural Networks and Activity Recognition

In the past few years, CNNs have been applied in a variety of manners to HAR, in both the fields of ambient and wearable sensing. Mo et al applied a novel approach utilizing machine vision methods to recognize twelve daily living tasks with the Microsoft Kinect. Rather than extract features from the Kinect data streams, they developed 144×48 images using 48 successive frames from skeleton data and 15×3 joint position coordinates and 11×3×3 joint rotation matrices. These images were then used as input to a multilayer CNN which automatically extracted features from the images that were fed in to a multilayer perceptron for classification [21]. Stefic and Patras utilized CNNs to extract areas of gaze fixation in raw image training data as participants watched videos of multiple activities [22]. This produced strong results in identifying salient regions of images that were then used for action recognition. Ma et al also combined a variety of CNNs to complete tasks, such as segmenting hands and objects from first-person camera images and then using these segmented images and motion images to train an action-based and motion-based CNN [23]. This novel use of CNNs allowed an increase in activity recognition rates of 6.6%, on average. These research efforts demonstrated the power of utilizing CNNs in multiple ways for HAR.

Research utilizing CNNs for HAR with wearable inertial sensors has also been published recently. Zeng et al implemented a method based on CNNs which captures the local dependency and scale invariance of an inertial sensor signal [24]. This allows features for activity recognition to be identified automatically. The motivation for developing this method was the difficulties in identifying strong features for HAR. Yang et al also highlighted the challenge and importance of identifying strong features for HAR [25]. They also employed CNNs for feature learning from raw inertial sensor signals. The strength of CNNs in HAR was again demonstrated here as its use in this circumstance outperformed other HAR algorithms, on multiple datasets, which utilized heuristic hand-crafting of features or shallow learning architectures for feature learning. Radu et al also recently demonstrated that the use of CNNs to identify discriminative features for HAR when using multiple sensor inputs from various mobile phones and smartwatches, which have different sampling rates, data generation models, and sensitivities, outperforms classic methods of identifying such features [26]. The implementation of such feature learning techniques with CNNs is clearly beneficial but is complex and may not be suitable for HAR system developers without strong experience in machine learning and DSP. From a CNN perspective, these results are interesting and suggest significant scope for further exploration for machine learning researchers. However, for the purposes of this paper, their inclusion is to both succinctly acknowledge that CNN has been applied to HAR previously and to distinguish the present approach which seeks to use well developed CNN platforms tailored for machine vision tasks in a transfer learning context for HAR recognition using basic time series as the only user created features.

#### Transfer Learning in Machine Vision

Deep learning-based machine vision techniques are used in many disciplines, from speech, video, and audio processing [27], through to HAR [21] and cancer research [28].

Training deep neural networks is a time consuming and resource intensive task, not only needing specialized hardware (graphics processing unit [GPU]) but also large datasets of labeled data. Unlike other machine learning techniques, once the training work is completed, querying the resulting models to predict results on new data is fast. In addition, trained networks can be repurposed for other specific uses which are not required to be known in advance of the initial training [29]. This arises from the generalized vision capabilities that can emerge with suitable training. More precisely, each layer of the network learns a number of features from the input data and that knowledge is refined through iterations. In fact, the learning that happens at different layers seems to be nonspecific to the dataset, including the identification of simple edges in the first few layers, the subsequent identification of boundaries and shapes, and growing toward object identification in the last few layers. These learned visual operators are applicable to other sets of data [30]. Transfer learning then is the generic name given to a classification effort when a pretrained network is reused for a task for which it was not specifically trained for. Deep learning frameworks such as Caffe [31] and TensorFlow can make use of pretrained networks, many of which have been made available by researchers in repositories such as the Caffe Model Zoo, available in their github repository.

Retraining requires not only a fraction of the time that a full training session would need (min/h instead of weeks), but more importantly in many cases, allows for the use of much smaller datasets. An example of this is the inception model provided by Google, whose engineers reportedly spent several weeks training on ImageNet [32] (a dataset of over 14 million images in over 2 thousand categories), using multiple GPUs and the TensorFlow framework. In their example [33], they use in the order of 3500 pictures of flowers in 5 different categories to retrain the generic model, producing a model with a fair accuracy rating on new data. In fact, during the retraining stage, the network is left almost intact. The final classifier is the only part that is fully replaced, and “bottlenecks” (the layer before the final one) are calculated to integrate the new training data into the already “cognizant” network. After that, the last layer is trained to work with the new classification categories. This happens in image batches of a size that can be adapted to the needs of the new dataset (alongside other hyperparameters such as learning rate and training steps).

Each step of the training process outputs values for training accuracy, validation accuracy, and cross entropy. A large difference between training and validation accuracy can indicate potential “overfitting” of the data, which can be a problem especially with small datasets, whereas the cross entropy is a loss function that provides an indication of how the training is progressing (decreasing values are expected).

## Methods

### Study Design

**Figure 2:**
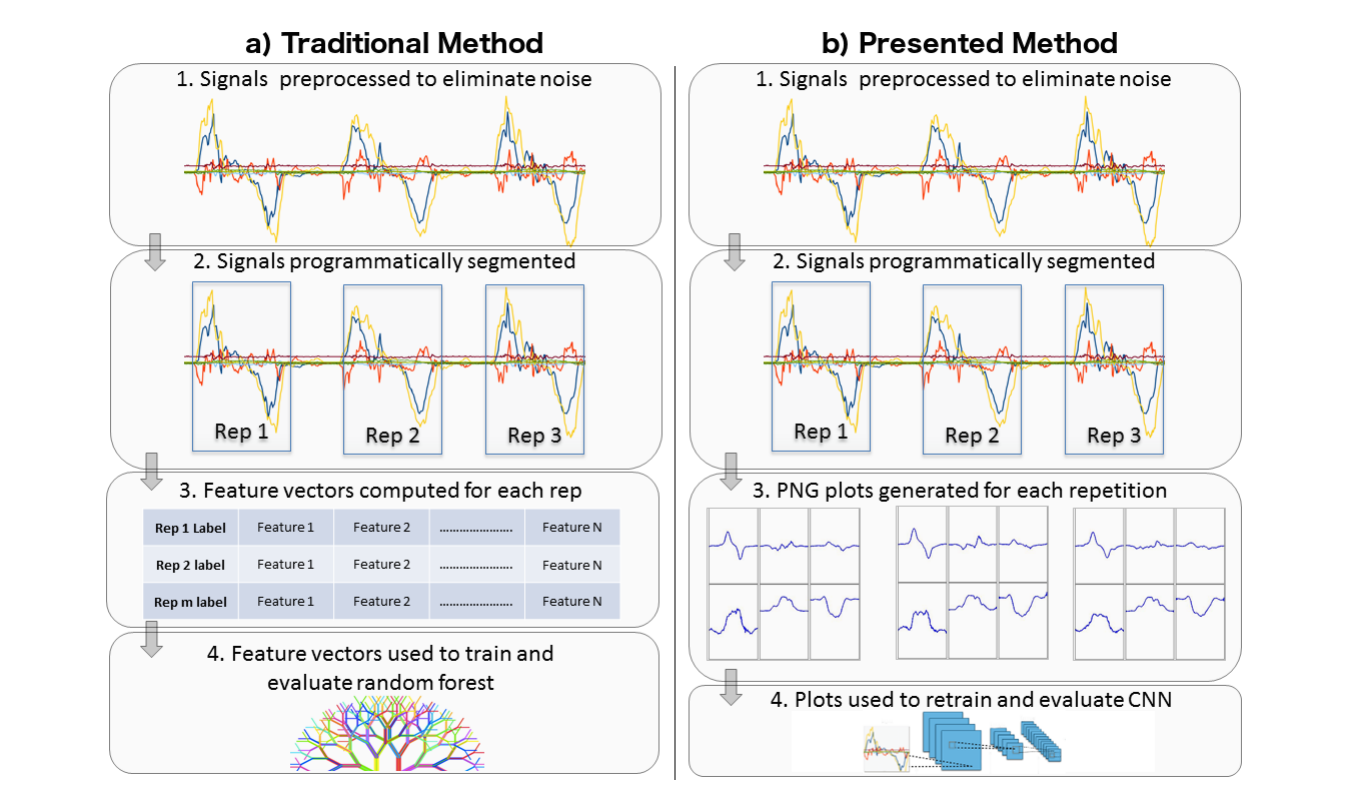
Depiction of the changes between traditional methods and the one presented in this paper, in particular steps 3 and 4.

Given the potential advantages of transfer learning in machine vision for the purposes of HAR, we next describe an exemplar study where we apply these ideas for the purposes of classifying exercise data from inertial sensors. This very specific example is sufficiently comprehensive in scale, and scope to represent a typical use case for the approach which to reiterate will use pretrained CNNs with one lightweight additional training step, to classify inertial sensor data based on images generated from the raw data (Figure 2). The level of DSP skills to perform this analysis will be shown to be much lower compared with other methods of classifying this type of data with other machine learning techniques that rely on engineered features (Figure 1).

This section contains all the details required to replicate this approach, focusing on how the data was collected, and how our system was set up and used.

### Data Collection

#### Participants

A total of 82 healthy volunteers aged 16-38 years (59 males, 23 females, age: 24.68 years [SD (standard deviation) 4.91], height: 1.75m [SD 0.09], body mass: 76.01kg [SD 13.29]) were recruited for the study. Participants did not have a current or recent musculoskeletal injury that would impair performance of multi-joint, lower-limb exercises. All participants had been completing each of the five exercises as part of their training regime for at least one year. The human research ethics committee at University College Dublin approved the study protocol and written informed consent was obtained from all participants before testing. In cases where participants were under the age of 18 years, written informed consent was also obtained from a parent or guardian.

#### Procedures

**Figure 3:**
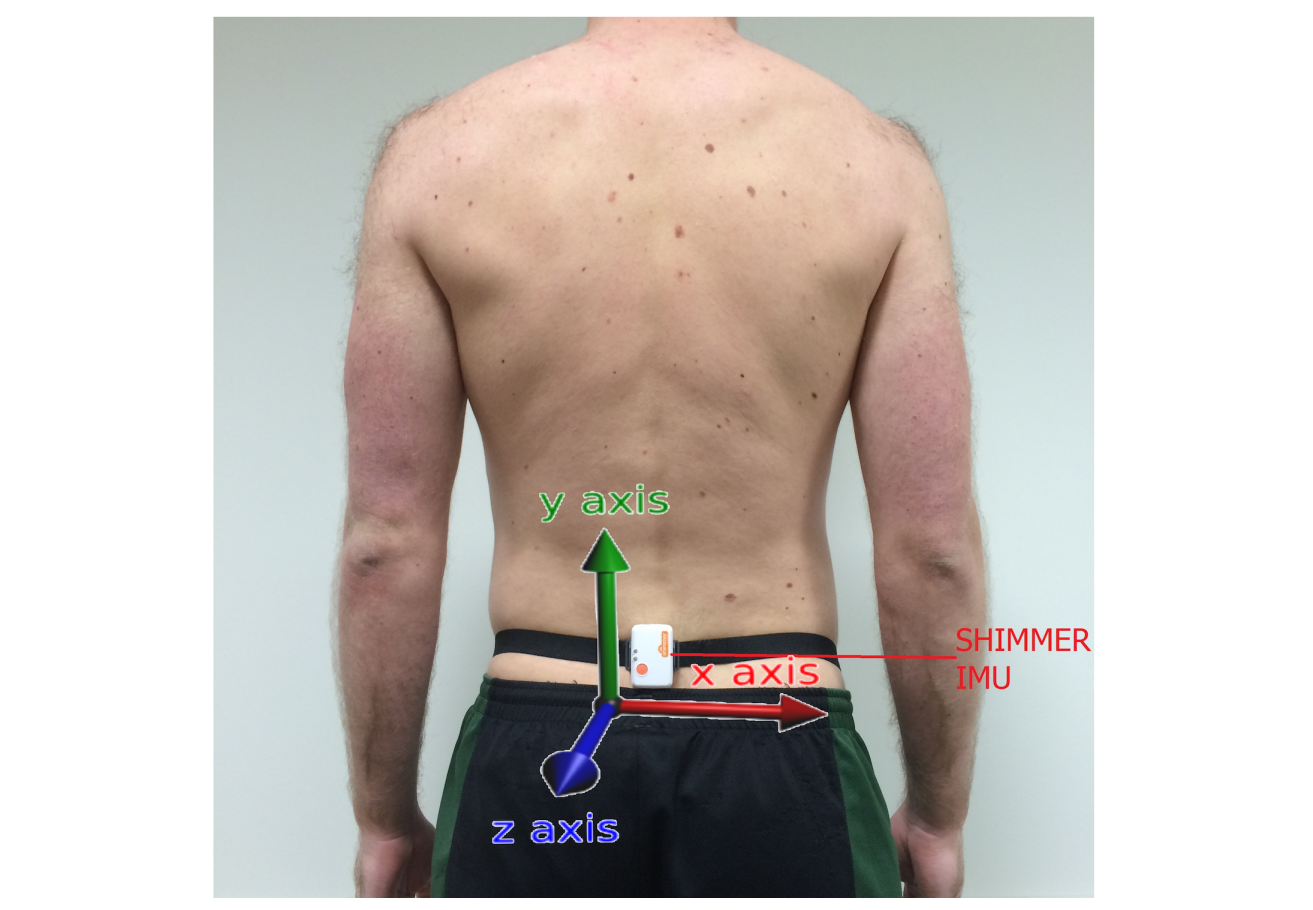
Inertial measurement unit (IMU) position: the spinous process of the 5th lumbar vertebra.

The testing protocol was explained to participants upon their arrival at the laboratory. Following this, they completed a 10-min warm-up on an exercise bike (Lode BV, Groningen, The Netherlands), maintaining a power output of 100W at 75-85 revolutions per min. Next, an IMU (SHIMMER, Dublin, Ireland) was secured on the participant by a chartered physiotherapist at the spinous process of the 5th lumbar vertebra (Figure 3). The orientation and location of all the IMUs was consistent for all the study participants across all exercises.

A pilot study was used to determine an appropriate sampling rate and the ranges for the accelerometer and gyroscope on board the IMU. In the pilot study, squat, lunge, deadlift, single-leg squat, and tuck jump data were collected at 512 samples/s. A Fourier transform was then used to determine signal and noise characteristics of the signal that were all found to be less than 20 Hz. Therefore, a sampling rate of 51.2 samples/s was deemed appropriate for this study based upon the Shannon sampling theorem and the Nyquist criterion [34]. The Shimmer IMU was configured to stream tri-axial accelerometer (±16 g) and gyroscope (±500 ˚/s) data with the sensor ranges chosen based upon data from the pilot study. Each IMU was calibrated for these specific sensor ranges using the Shimmer 9DoF Calibration application.

**Figure 4:**
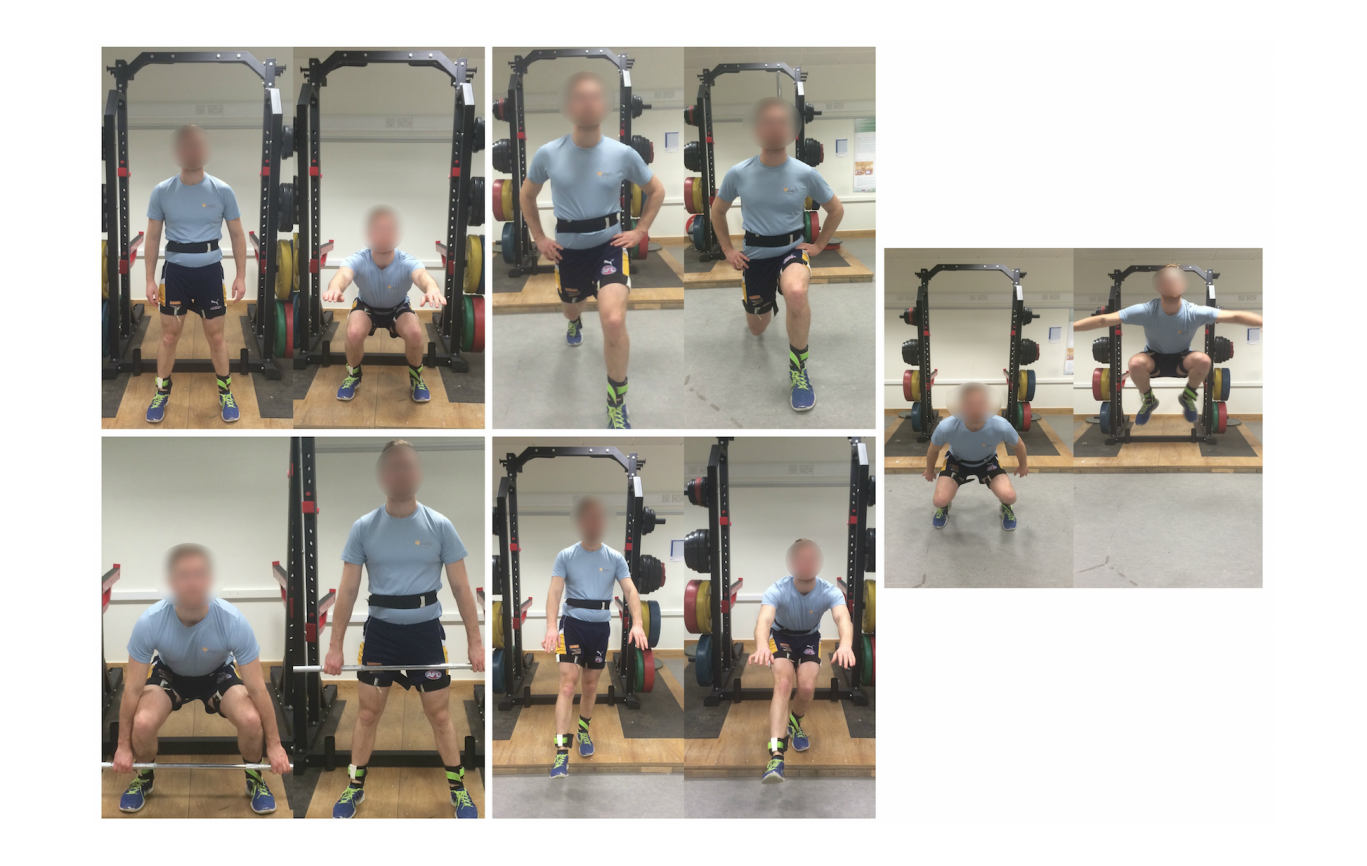
The five exercises completed for this study: bodyweight squat (upper left), bodyweight lunge (upper middle), barbell deadlift (lower left), single leg squat (lower middle), and tuck jump (right).

After completion of their warm up, participants proceeded to do one set of 10 repetitions of bodyweight squats, barbell deadlifts at a load of 25kg, bodyweight lunges, and bodyweight single-leg squats (Figure 4). A chartered physiotherapist demonstrated the correct technique for each of the exercises. Participants familiarized themselves with each exercise, and their technique was assessed to be correct by the physiotherapist. Correct technique for squats, lunges, and deadlifts was defined using guidelines from the National Strength and Conditioning Association [35]. Single leg squats were completed according to the scoring criteria outlined by Whatman et al [36]. Finally, each participant completed the 10-second tuck jump test while attempting to maintain good form throughout [37].

### Preparation for Transfer Learning

Based on the previous design for an IMU-based exercise classification system (Figure 1), with this new method the feature extraction step is not needed (Figure 2) as the CNN will take care of automatically both training the model and discovering the features by itself. The segmentation process is directly followed by the classification task (training and inference).

#### Convolutional Neutral Network (CNN) Infrastructure

Working with convolutional networks is not a trivial task. Fortunately, since the advent of deep learning in the last few years, a number of frameworks such as TensorFlow and Caffe have appeared in the market and are readily available for researchers. Most of these frameworks are open source, supported by large companies or universities, and provide not only helper libraries for numerical computation and machine learning but also a flexible architecture and the possibility to almost trivially use multiple central processing units (CPUs) and GPUs if available.

The authors used TensorFlow for the particular results provided in this paper, but any other framework or higher level library would suffice. Installing TensorFlow can be cumbersome, but Google provides a Docker container [38] with all the components to run TensorFlow out of the box. Documentation and scripts are also provided to retrain [39] networks and query [40] the new classifier. The aforementioned Docker container and scripts were used in this paper with minimal modifications.

The preprocessing and segmentation of inertial data to create the images that are fed into the CNN were prepared with MATLAB (2012, The MathWorks), as explained in the following section.

#### Data Preparation

Six signals were collected from the IMU; accelerometer *x, y, and z;* and gyroscope *x, y, and z*. Data were analyzed using MATLAB. To ensure the data analyzed applied to each participant’s movement and to eliminate unwanted high-frequency noise, the six signals were low pass filtered at f_c_=20 Hz using a Butterworth filter of order n=8.

**Figure 5:**
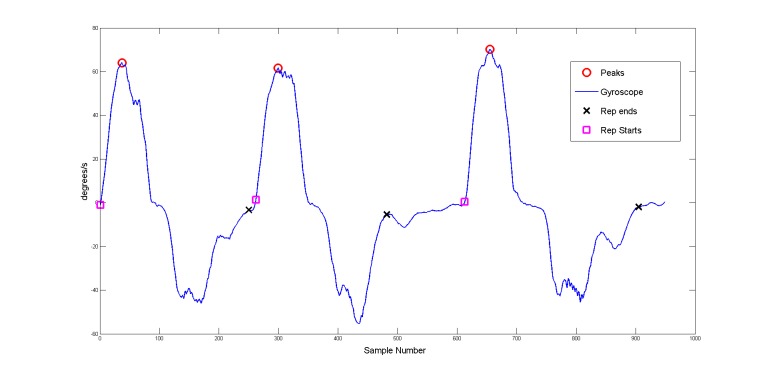
Detection of peak, start, and end points of exercise repetitions (neighboring zero crossing values to the peak locations).

The filtered signals were then programmatically segmented into epochs that relate to single, full repetitions of the completed exercises. Many algorithms are available to segment human motion during exercise. These include the sliding window algorithm, top-down, bottom-up algorithms, zero-velocity crossing algorithms, template-base matching methods, and combination algorithms of the above [4]. These algorithms all have advantages and disadvantages. For the purpose of the creation of a functioning exercise detection classifier, a simple peak-detection algorithm was used on the gyroscope signal with the largest amplitude for each exercise. The start and end points of each repetition were found by looking for the corresponding zero-crossing points of the gyroscope signal leading up to and following the location of a peak in the signal. Example results of the segmentation algorithm used on the gyroscope *x* signal, from an IMU positioned on the spine during 3 repetitions of the deadlift exercise, are provided (Figure 5).

**Figure 6:**
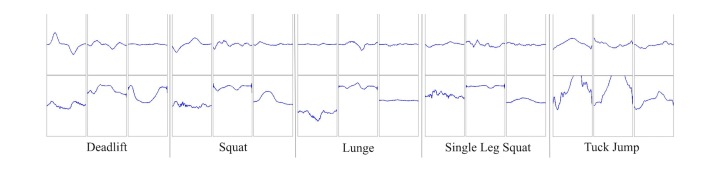
Samples of the generated plots (JPEG files) which were used as training and test data in this study.

Each extracted repetition of exercise data was resampled to a length of 250 samples. The six signals were then plotted using the MATLAB subplot function. The first subplot, gyroscope *x* (sagittal plane) was plotted between the y-axis range of ±250 °/s. Subplots 2 and 3, gyroscope *y* and *z* (frontal and transverse plane) were plotted between the y-axis range of ±100 °/s. Accelerometer *x* (subplot 4) was plotted in the y-axis range of ±3 m/s^2^ and accelerometer *y* and *z* (subplots 5 and 6) were plotted in the range ±15 m/s^2^. Axes labels and markers were programmatically hidden, and the blank space between each subplot was minimized. Following this, the graphs were saved as 470x470 JPEG files. Examples of the generated JPEG files are provided (Figure 6).

#### Retraining and Using the New Model

**Figure 7:**
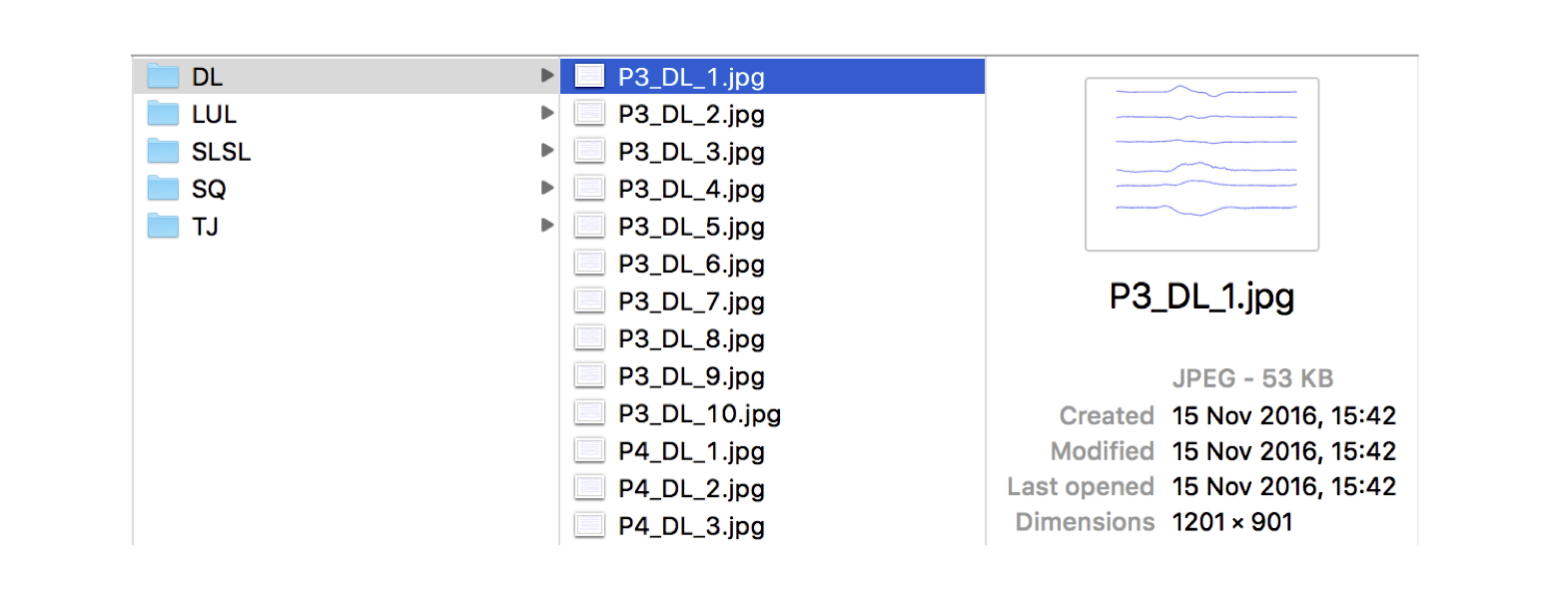
Folders containing images for the five exercises (Bodyweight squat: SQ, bodyweight lunge: LUL, barbell deadlift: DL, single leg squat: SLSL, and tuck jump: TJ).

Transfer learning is the main technique used in this paper. This reuses an already trained CNN for classification purposes. In this case, the framework TensorFlow was used, which provides access to a model called “inception” trained on over 14 million images and also provides example scripts to retrain the network, that is, discarding the provided classifier and adjusting the values of the last layer of the network according to the new data provided. The retraining scripts expect to find the images in a particular folder (passed as a parameter) and layout (Figure 7), that is, a folder for each category that the new classifier will learn to identify, containing training pictures in jpg format. During training, the network will automatically identify the features to use to create the classifier.

There are a number of hyperparameters that can be changed depending on the new data used to retrain, such as the validation and training split of data to be used, the size of the batches to train on, or the learning rate applied (probably the most important of all for fine tuning and avoiding extra computation). The only parameter changed in this work was the number of steps, from a default 4000 iterations to 96,000 steps. This number provides high accuracy without showing signs of overfitting (see Results section).

The output of the training phase is simply two files, one with new weights (the retrained network) and a second file with labels for the data trained (the default names are retrained_graph.pb and retrained_labels.txt). These two files are all that is needed to predict results coming from new data. The classifier can be queried with the classify_image script mentioned previously.

Retraining and querying are actions that can be performed in a multitude of ways, with different frameworks and in different configurations. This work is about making things accessible and available. The Docker container for Tensorflow, with the documentation and helper scripts, was the simplest route the authors could find.

## Results

**Figure 8:**
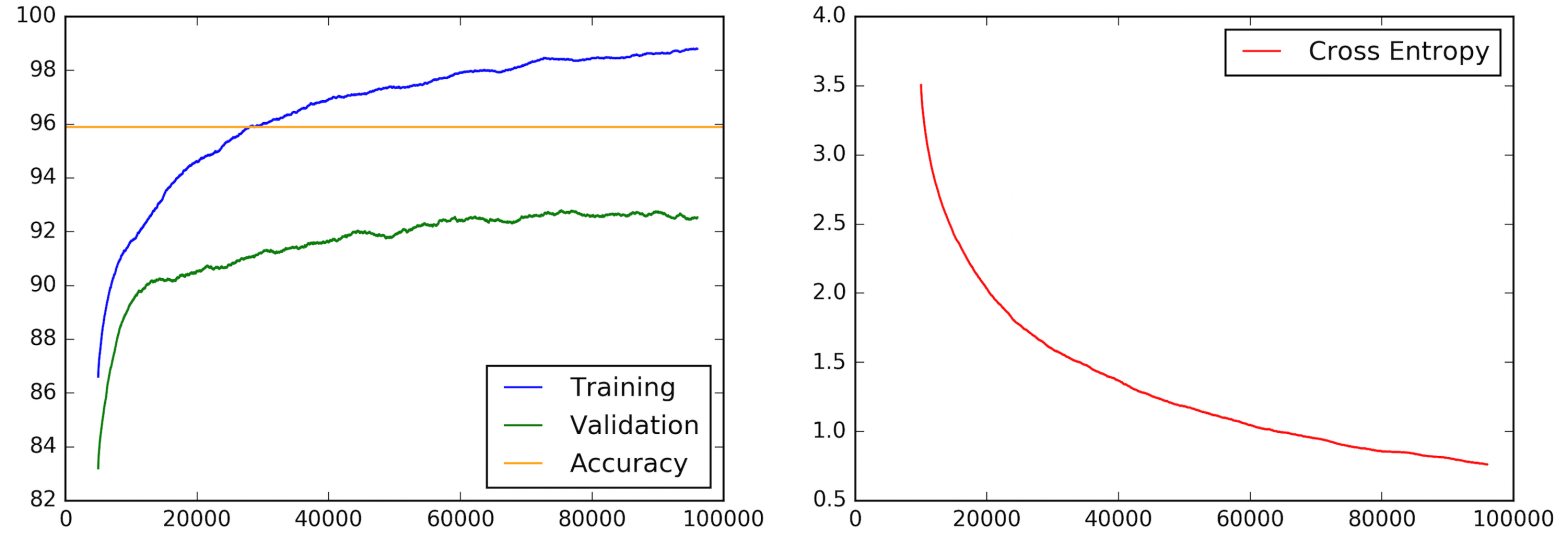
Training (blue) and validation (green) accuracy during training phase, with final accuracy (orange) and cross entropy (red) for 96,000 steps.

As mentioned in the previous section, each training batch outputs training and validation accuracy and a cross entropy (loss function) amount, alongside with final validation accuracy. Rolled averages for those four values for training sessions of 96,000 steps are shown (Figure 8).

**Figure 9:**
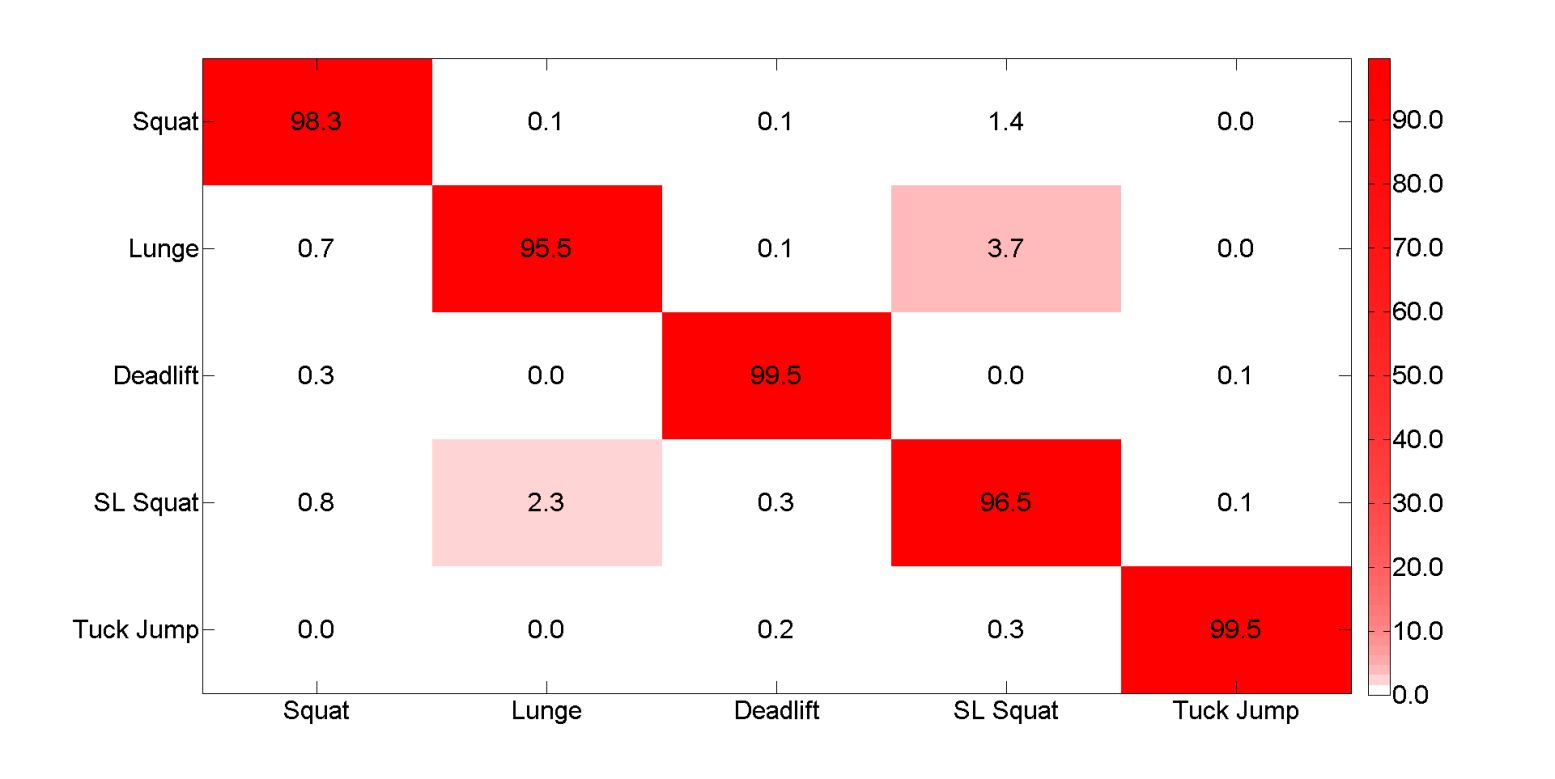
Confusion matrix for the machine vision-based classification method.

As observed, the cross entropy keeps falling steadily, and the average difference between training and testing is not very large, so overfitting is not an issue. Averaged over 5 runs of training, the final accuracy result was a 95.89% (3928/3991) for 96,000 steps. Figure 9 shows a confusion matrix for this method.

**Figure 10:**
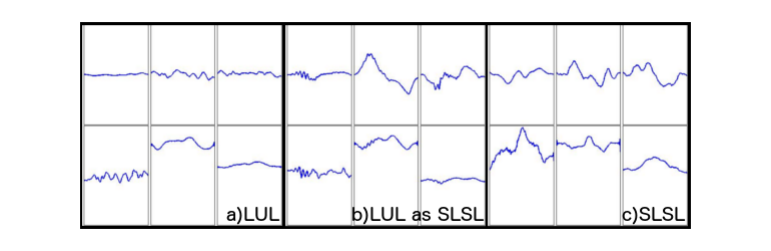
A lunge signal (a), a lunge signal misclassified as a single leg squat (b), and a single leg squat signal (c) for comparison.

Figure 10 is an illustration of a misclassified plot. Part (a) of the image shows a typical lunge signal, whereas part (c) shows a typical single leg squat signal. Part (b) in the middle shows an example of a lunge repetition misclassified as a single leg squat. The issue seems to be concentrated in the top part of the image. The most likely reason for the odd lunge signal shape is that the subject may have looked over their shoulder or twisted for some reason during the repetition, and the final result is confusing the classifier, as it would confuse an expert looking directly at the plot.

**Figure 11:**
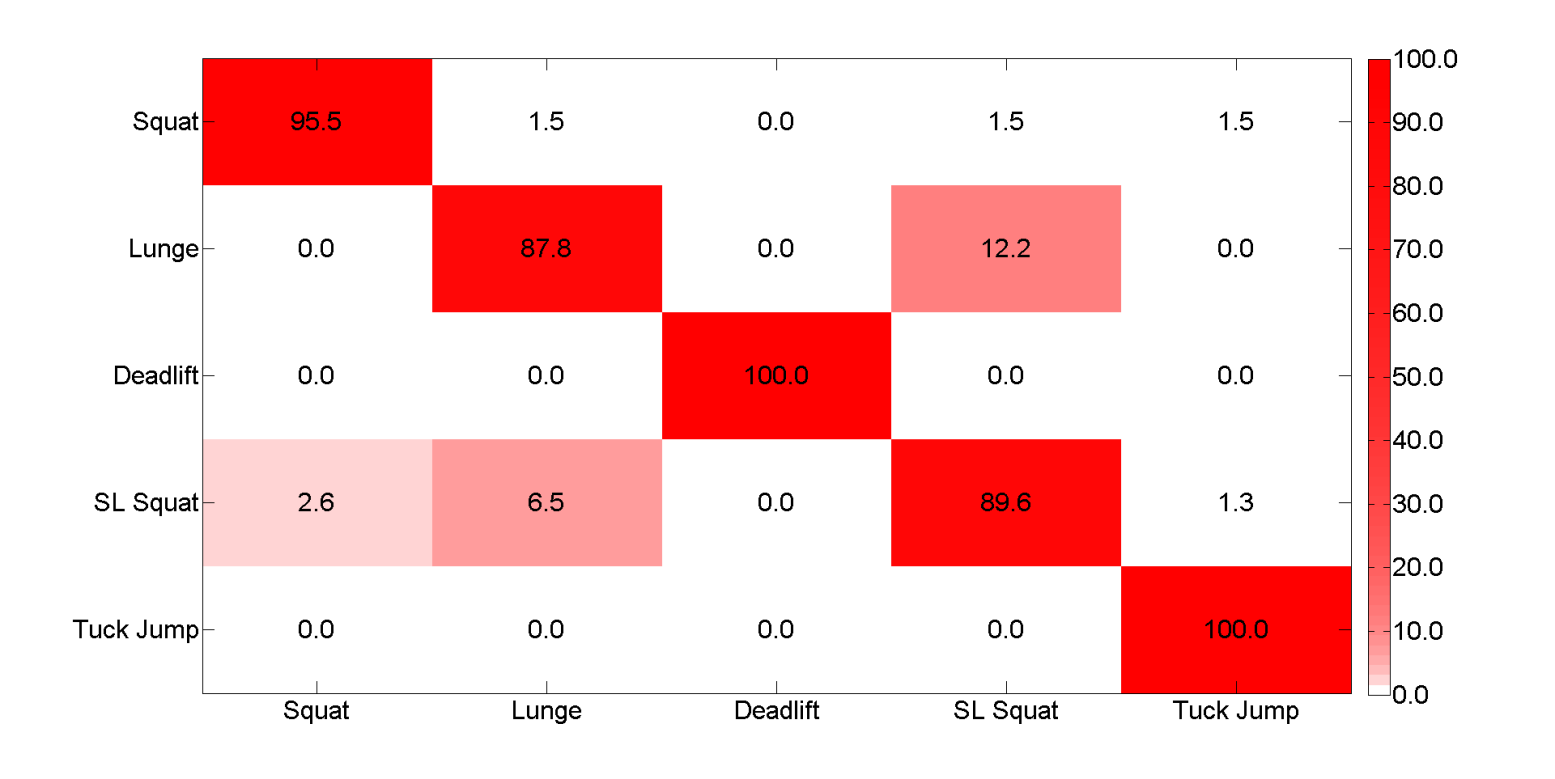
Confusion matrix for the feature based classification method.

These results are equivalent with a recently published method on the same dataset whereby the accuracy was found to be 94.1% [17]. Figure 11 shows the confusion matrix for this feature-based classification effort, and as it can be seen, the results are similar. However, leave-one-subject-out-cross- validation was used in this instance, so the results are not directly comparable.

The emphasis on this work, though, is in the ease of setup by using transfer learning and the need of only basic digital processing skills to prepare the data, when compared with other methods in this area.

## Discussion

### Principal Findings

An analysis of the data collected with the method proposed obtained an average 95.89% (3827/3991) classification accuracy, which is competitive with current state-of-the-art techniques. This high level of accuracy indicates that the distinctive waveforms in the plots for each of the exercises can be generalized among different participants, and the patterns created are appropriate for classification efforts. These results are coupled with the underlying recurrent theme for this work—to enable a more approachable entry path into the HAR and ED fields. To do so, high-level machine learning frameworks, coupled with a novel use of machine vision techniques, are used in two main ways: first, to avoid the complexity of manually crafted features only available through advanced DSP techniques, and second, to facilitate dimensionality reduction by allowing the CNN to take care of both feature extraction and classification tasks.

### Comparison With Prior Work

The methodology employed and the results achieved in this paper can be directly compared with a recently published ED paper on the exact same dataset [17]. In this recently published work, identical filtering and segmentation methodologies were employed. However, a vast amount of additional signals and data processing were required to achieve classification with the lumbar worn IMU. As well as the 6 signals from the accelerometer and gyroscope used in this paper, 12 additional signals were used for classification. These were magnetometer *x, y,* and *z,* magnitude of acceleration, magnitude of rotational velocity, and the IMU’s three-dimensional (3-D) orientation as represented by a rotation quaternion (W, X, Y, and Z) and Euler angles (pitch, roll, and yaw). Furthermore, 19 features were then computed from the segmented epochs of the 18 signals. These features were namely “mean,” “RMS,” “standard deviation,” “kurtosis,” “median,” “skewness,” “range,” “variance,” “max,” “index of max,” “min,” “index of min,” “energy,” “25th percentile,” “75th percentile,” “level crossing rate,” “fractal dimension,” and the “variance of both the approximate and detailed wavelet coefﬁcients using the Daubechies 4 mother wavelet to level 7.” This resulted in a total of 342 features per exercise repetition. These features and their associated exercise label were used to evaluate and train a random forests classifier with 400 trees. Following leave-one-subject-out-cross-validation an accuracy result of 94.64% was achieved with this method. This recent work also demonstrated the laborious process of identifying the most important features for classification that can improve the efficiency of the reported technique used.

Although the accuracy result achieved in this recent work (94.64%) is slightly less than that presented in this paper (95.89%), the results should not be directly compared. This is because the additional signals used by O’Reilly et al [17] and the different method of cross-validation utilized in both studies to compute accuracy mean it is not a perfectly like-for-like comparison. However, it can be stated that similar levels of accuracy have been achieved with both methods. Most importantly, the ease of implementation of the classification method presented here greatly exceeds that presented by O’Reilly et al [17]. Most notably, the need to use additional signals and derive many features from them has been eliminated. This minimizes the signal processing and machine learning experience needed by the person investigating the possibility of creating a classifier. This is in line with the core objective of this paper.

### Limitations

Simplicity was of utmost importance when designing this novel classification method for accelerometer and gyroscope data. Subsequently, maximal possible accuracy may not have been achieved. Utilizing a better understanding on how to parameterize the retraining effort and other techniques such as fine tuning (a method to reuse certain parts of a pretrained network instead of simply changing the last layer and classifier), could produce better results. A better understanding on how to deal with the type of data we are using could be beneficial. In general, machine vision work is plagued with issues such as partial occlusion, deformation, or viewpoint variation, which the data in this work does not suffer from. Due to that, and also to make the baseline of this work as simple as possible, no data augmentation or any kind of image processing techniques has been used. The results reported have been obtained only with resources from readily available frameworks, mostly on default settings.

It should also be noted that the presented method of classifying inertial sensor data with machine vision techniques has only been evaluated on exemplar samples of exercises that were conducted in a laboratory setting. Results are of high accuracy and competitive, with recent work on the same dataset [17] and therefore, act as a proof of concept for the method. However, the method has not yet been evaluated in classifying inertial sensor data arising from free-living activities and other HAR classification tasks. Future work should investigate the method’s efficacy in such areas. Of key importance will be to simplify each application’s preprocessing and segmentation of the inertial sensor data.

### Conclusions

This paper has described a novel application approach for the classification of inertial sensor data in the context of HAR. There are two stand-out benefits of the machine vision approach described. The first is the ease of setting up the infrastructure for the CNNs involved through the use of transfer learning. The second is the reduction in the depth of digital signal processing expertise required on the part of the investigator. Due to the many difficulties in creating inertial sensor based activity recognition systems, the authors believe there is a need for a system development path which is easier to use for people who lack significant background in signal processing and machine learning. In particular, the new development pathway should eliminate the most difficult tasks conventionally identified with this area, that is, feature development or extraction and dimensionality reduction for the best machine learning method for each new application (Figure 1). The new development pathway, although eliminating these steps, does not compromise the attainment of high quality classification accuracy, sensitivity, and specificity which is currently achieved through their successful implementation by appropriate experts (Figure 4). The exemplar study described here illustrates that the method is very competitive in comparison with customized solutions. Either way, the new pathway, at the very least, will allow for the easier testing of hypotheses relating to new inertial sensor-based activity classification systems, that is, is the classification possible at all based on the collected dataset? Ideally, it should also achieve equivalent.

Whereas the presented method does successfully eliminate the need for feature crafting and identification of optimal classification algorithms, it does not eliminate the process of signal preprocessing and signal segmentation before performing classification. Therefore, there remains some complexity in the process of achieving exercise classification when using the machine vision technique. However, the authors consider the process of filtering, segmenting, and plotting inertial sensor signals considerably less complex than identifying and computing strong features and an optimal classification method for the classification of inertial sensor data.

### Future Work

Even though the current infrastructure used is readily available, certain skills such as familiarity with Docker or with Python data science stacks and basic DSP skills are still needed. The creation of a full package that could be installed on the researcher’s machine could be an avenue to explore. Also the preprocessing and segmentation steps to prepare the data could be simplified by providing a set of scripts.

A number of professional machine vision companies exist in the market, and some provide online services that allow retraining of their custom models and could also be used for this type of work, avoiding the need for setting up the CNN infrastructure locally.

The availability of this technology on Android mobile devices is something that the authors are also pursuing. TensorFlow may provide some initial support in this area. Finally, although this paper emphasizes the lack of a necessity to present features other than the basic time series, it is clear that augmentation with derived features presents further opportunities for performance tweaking. For researchers more comfortable with such feature development, this application avenue is worth exploring.

## Abbreviations

3-D: three-dimensional
CNN: convolutional neural network
CPU: central processing unit
DSP: digital signal processing
ED: exercise detection
GPU: graphics processing unit
HAR: human activity recognition
IMU: inertial measurement unit
SD: standard deviation
